# Diagnostic Performance of a Large Language Model for Determining the Cause of Death: A Comparative Analysis of Clinical History, Postmortem Computed Tomography Findings, and Their Integration

**DOI:** 10.7759/cureus.83721

**Published:** 2025-05-08

**Authors:** Masanori Ishida, Wataru Gonoi, Keisuke Nyunoya, Hiroyuki Abe, Go Shirota, Naomasa Okimoto, Kotaro Fujimoto, Mariko Kurokawa, Akira Katayama, Masumi Takahashi-Mizuki, Shohei Inui, Kazuhiro Saito, Tetsuo Ushiku, Osamu Abe

**Affiliations:** 1 Radiology, Tokyo Medical University Hospital, Tokyo, JPN; 2 Radiology, The University of Tokyo Hospital, Tokyo, JPN; 3 Pathology, The University of Tokyo Hospital, Tokyo, JPN

**Keywords:** artificial intelligence, cause of death, claude 3.5 sonnet, large language model, postmortem computed tomography (pmct)

## Abstract

Introduction: This study evaluates the diagnostic performance of a large language model (LLM) in determining causes of death by comparing three different information sources.

Methods: A total of 150 consecutive adult in-hospital cadavers underwent postmortem CT and pathological autopsy (2009-2013). The diagnostic accuracy of Claude 3.5 Sonnet (Anthropic, San Francisco, California) was evaluated in determining both underlying and immediate causes of death using three different information sources (clinical history alone, postmortem CT findings alone as documented by radiologists in their reports, and their integration). For each case, the LLM provided a primary diagnosis and two differential diagnoses. The autopsy result was used as the reference standard to assess accuracy.

Results: For underlying causes, the integration of both sources achieved significantly higher accuracy (78.0%) compared with the clinical history alone (69.3%) or the CT findings alone (42.0%) (p<0.001). When considering either primary or differential diagnoses, the accuracy reached 84.7% with integrated sources, 78.0% with clinical history alone, and 58.7% with CT findings alone. For immediate causes, the integrated approach showed higher accuracy in the primary diagnosis (61.3%) than the clinical history alone (52.0%) and CT findings alone (46.7%) (p<0.001). Disease-specific diagnostic accuracy analyses revealed marked variations, with hematologic malignancies showing the most significant differences among information sources (clinical history: 78.9%, CT findings alone: 36.8%, integrated analysis: 85.7%; p=0.003).

Conclusion: Integrating postmortem CT findings with clinical history enhances LLM-based cause-of-death determination accuracy, demonstrating the value of multiple information sources while highlighting opportunities for disease-specific diagnostic optimization.

## Introduction

The accurate determination of the cause of death is crucial for both medical and legal purposes, requiring careful integration of clinical information and postmortem findings. Recent studies have demonstrated remarkable progress in the capabilities of large language models (LLMs) across various medical diagnostic tasks [1-11], including clinical reasoning, radiological interpretation, and complex case analysis. Although these advances are promising [12,13], LLM application to death investigation remains largely unexplored [14].

The field of postmortem diagnostics faces significant challenges, including declining autopsy rates worldwide and growing shortages of forensic pathologists and specialized radiologists trained in postmortem imaging interpretation. These challenges have increased interest in developing computer-assisted tools to support death investigation processes. While AI applications have shown promise in various medical domains, their potential to assist in determining causes of death remains largely unexplored, particularly regarding the integration of multiple information sources that experts typically consider.

Cause-of-death interpretation involves the following two distinct aspects: the underlying cause (the primary disease initiating the chain of events leading to death, such as lung cancer) and the immediate cause (the final condition resulting in death, such as respiratory failure) [14,15]. This dual nature of death certification presents unique challenges, requiring the integration of multiple information sources, including the patient's clinical history and postmortem imaging findings [15,16]. While radiologists routinely perform this integration based on their expertise, the potential for LLMs to facilitate this process warrants investigation, particularly given the growing shortage of specialists in death investigation [17].

Postmortem CT has become increasingly important as a complementary or alternative approach to conventional autopsy. However, its diagnostic accuracy varies considerably depending on the pathology involved and is often enhanced when combined with clinical information. Understanding how LLMs process and integrate these different information sources could provide insights into their potential utility as diagnostic support tools in forensic and clinical death investigations.

This study aimed to evaluate the diagnostic performance of an LLM in determining both underlying and immediate causes of death using three different information sources: clinical histories alone, postmortem CT findings alone, and their integration (a combination of both). We hypothesized that the integration of multiple information sources would improve diagnostic accuracy and that different information sources might show varying levels of utility across disease categories.

## Materials and methods

Study population

This retrospective study was approved by the institutional review board of our tertiary care center. Written informed consent for both postmortem CT and autopsy, including potential future research use of the data, was obtained from the families of the deceased at the time of death during the original study period (2009-2013). Between April 2009 and December 2013, 216 consecutive cases of in-hospital death at our facility underwent both postmortem CT and pathological autopsy. These cases represented patients who died during hospitalization and underwent autopsy for cause-of-death investigation. Of these, we excluded 12 cases of deceased people aged under 18 years, 15 cases with only the underlying cause of death unknown at autopsy, and 39 cases with either the underlying or immediate cause of death (or both) unknown at autopsy. Finally, 150 cases of adult in-hospital death with autopsy-confirmed causes of death were included in this study. We employed consecutive sampling, including all eligible adult in-hospital deaths that underwent both postmortem CT and pathological autopsy during the study period. This approach was chosen over probability sampling methods due to the retrospective nature of the study and the inherent limitations in obtaining cases with complete postmortem imaging and autopsy correlation. Sample size was not determined by formal power calculations but was based on the total available cases meeting our inclusion criteria, consistent with similar diagnostic performance studies in postmortem imaging. The resulting sample size of 150 cases allowed for meaningful statistical analysis of the primary outcomes while maintaining adequate representation across major disease categories. While our dataset spans from 2009 to 2013, this temporal gap does not significantly impact our study objectives, as our primary aim was to evaluate the LLM's diagnostic performance when provided with different information sources rather than to assess the diagnostic capabilities of postmortem CT technology itself. This historical cohort provided the advantage of complete clinical, imaging, and pathological autopsy correlation necessary for comprehensive evaluation of an LLM's diagnostic capabilities across multiple information sources.

Data collection and preparation

Two distinct levels of cause of death were analyzed as outcome measures: (1) the underlying cause of death and (2) the immediate cause of death [14,15]. We extracted both radiologists’ diagnoses from postmortem CT reports and pathologists’ final diagnoses from autopsy reports, with the latter serving as the reference standard.

Medical histories and postmortem CT findings of all cases were compiled after removing clinical and imaging diagnoses of the causes of death and personal information. Medical histories included the present illness at admission, clinical course during hospitalization, past medical history, social history, and family history. Detailed antemortem laboratory data, radiology reports, and pathology reports were not included. Postmortem CT reports were written independently of the medical history by two board-certified diagnostic radiologists with extensive postmortem imaging experience (10-17 years) in a blinded manner with regard to the autopsy findings.

Disease classification

The underlying causes of death were categorized into six major categories: malignant neoplasms, cardiovascular diseases, liver diseases, respiratory diseases, neurological diseases, and others.

The immediate causes of death were classified into six major categories: respiratory failure, circulatory failure, multiple organ failure, hepatic failure/gastrointestinal complications, neurological events, and others. This classification system was developed on the basis of previous studies of postmortem diagnosis [15,16,18].

For statistical analysis, cases with very small sample sizes (n≤2) were either integrated into larger relevant categories when appropriate (e.g., breast cancer (n=2) was included in the group of malignant neoplasms) or classified into the “others” category in the absence of a suitable larger category.

LLM analysis

We used the Claude 3.5 Sonnet (Anthropic, San Francisco, California), which was released on June 20, 2024, to list a primary diagnosis and two differential diagnoses of the underlying and immediate cause of death. When preparing the data for LLM analysis, we carefully excluded clinical and imaging diagnoses of the underlying and immediate causes of death, as well as personal information, from the input text. In defining the causes of death, we distinguished between two levels: the underlying cause of death and the immediate cause of death [14,15]. For each case, the LLM was required to provide a primary diagnosis and two additional differential diagnoses for both the underlying and immediate causes of death. We used the LLM to evaluate cases under three distinct conditions designed to assess the relative value of different information sources: (1) Clinical history only: "Please provide the likely underlying and immediate causes of death based on the patient’s medical history (including age, sex, and summary of medical history, excluding the clinical diagnosis), listing up to three possible causes for each in the order of likelihood." (2) Postmortem CT findings only: "Please provide the likely underlying and immediate causes of death based on the imaging findings on the postmortem CT report (including age and sex but excluding the summary of medical history, clinical diagnosis, and postmortem imaging diagnosis), listing up to three possible causes for each in the order of likelihood." (3) Integrated information: "Please provide the likely underlying and immediate causes of death based on the patient’s medical history and the imaging findings on the postmortem CT report (including age, sex, and summary of medical history, excluding the clinical diagnosis and postmortem imaging diagnosis), listing up to three possible causes for each in the order of likelihood."

**Figure 1:**
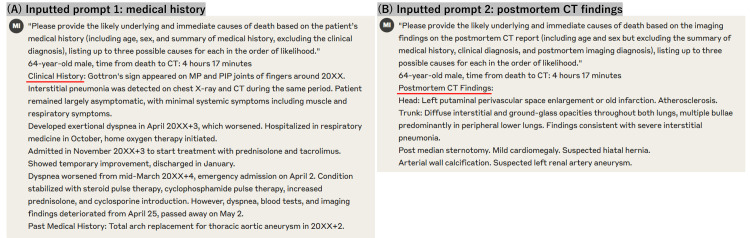
Question for prompt 1 and 2 samples (A) Manually typed question for prompt 1, age, sex, time from death to CT, and medical history; (B) Manually typed question for prompt 2, age, sex, time from death to CT, and postmortem CT findings The language used in the study is Japanese, but this example is shown in English.

To clarify, we provided the LLM with the text of the postmortem CT reports written by radiologists, not the CT images themselves, as Claude 3.5 Sonnet is a text-based model without the capability to directly interpret radiological images. All medical histories and CT reports remained in their original Japanese format, and the textual data were passed to the LLM without translation. Each condition was analyzed in a separate thread to prevent cross-contamination between analyses (Figure 1).

**Figure 2:**
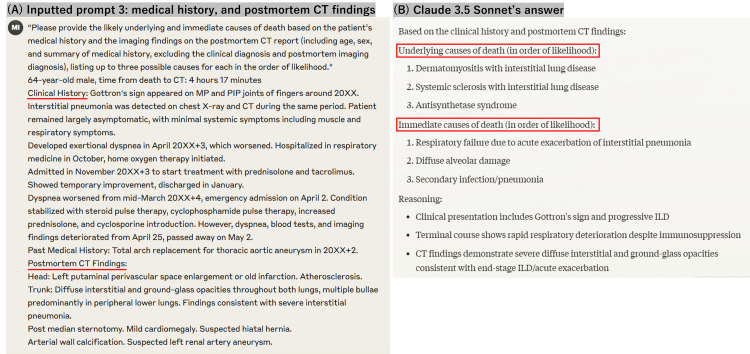
Question for prompt 3 and the response samples (A) Manually typed question for prompt 3, age, sex, time from death to CT, medical history, and postmortem CT findings; (B) Response generated by Claude 3.5 Sonnet The language used in the study is Japanese, but this example is shown in English. In this case, the underlying cause of death was dermatomyositis, and the immediate cause of death was respiratory failure due to acute exacerbation of interstitial pneumonia, consistent with the diagnoses from the autopsy.

The LLM provided a primary diagnosis and two differential diagnoses for both underlying and immediate causes in the order of likelihood (Figure 2).

To ensure reproducibility of our results, we fixed the LLM's generation parameters with temperature = 0.0 and top-p = 1.0. These settings eliminate randomness in the model's outputs, ensuring that repeated queries with identical inputs produce consistent responses.

Reference standard and diagnostic evaluation

The autopsy findings served as the reference standard. All autopsies were performed by board-certified pathologists immediately after postmortem CT. The pathological autopsies were performed according to our institution's standard protocol, where autopsies were conducted by pathologists who had access to the patients' clinical histories but did not routinely review the postmortem CT findings. The postmortem CT reports were prepared independently by radiologists without knowledge of the subsequent autopsy findings. This separation in the original clinical workflow allowed us to retrospectively analyze these as independent data sources, even though our study design itself was retrospective. Disease-specific diagnostic accuracy analyses examined both primary and the top three differential diagnoses. The LLM diagnostic accuracy was determined by consensus of two board-certified diagnostic radiologists with extensive postmortem imaging experience (15 and 17 years). Diagnoses were considered accurate if they exactly matched or were deemed synonymous with autopsy diagnoses. Ambiguous or insufficient responses were considered incorrect.

For analyzing the complementary performance of different information sources, "complete agreement" was defined as cases in which both diagnostic approaches (clinical history alone and CT findings alone) independently included the correct diagnosis within their top three differential diagnoses. Cases were classified into four categories: complete agreement (both sources correct within the top three), incorrect clinical history (clinical history incorrect within the top three but with correct CT findings), incorrect CT findings (CT findings incorrect within the top three but clinical history correct), and neither correct (both sources incorrect within the top three).

Statistical analysis

Quantitative variables are presented as mean values and standard deviations (for normally distributed data) or median values and interquartile ranges (for non-normally distributed data). Categorical variables are presented as frequencies and percentages. We employed chi-square tests for large-sample comparisons and Fisher’s exact test for smaller subgroups. Cochran’s Q test with the post-hoc McNemar’s test (Bonferroni correction) compared the three information sources. The inter-rater agreement used Cohen’s kappa. Disease-specific diagnostic accuracy analyses examined both the primary and top three differential diagnoses. The threshold for statistical significance was set at p<0.05 (two-sided). Analyses were performed using SPSS Version 29.0 (IBM Corp, Armonk, NY).

## Results

Study population and disease categories

The final study population consisted of 150 autopsy cases (104 men and 46 women; age range: 18-98 years, mean age: 66±14.8 years). Among these cases, the underlying causes of death were categorized into six major groups: malignant neoplasms (79 cases, 52.7%), cardiovascular diseases (24 cases, 16.0%), liver diseases (16 cases, 10.7%), respiratory diseases (7 cases, 4.7%), neurological diseases (11 cases, 7.3%), and others (13 cases, 8.7%).

The “others” category included autoimmune/inflammatory diseases (dermatomyositis (n=2), systemic sclerosis (n=1)), metabolic/systemic diseases (alkaptonuria (n=1), amyloidosis (n=2)), acute pancreatitis (n=1), Erdheim-Chester disease (n=1), bone marrow failure with miliary tuberculosis (n=1), liver allograft failure (n=1), gastrointestinal perforation (n=1), renal failure (n=1), and pulmonary vascular tumor (n=1).

The immediate causes of death were classified into six major categories: respiratory failure (72 cases, 48.0%), circulatory failure (24 cases, 16.0%), multiple organ failure (22 cases, 14.7%), hepatic failure/gastrointestinal complications (15 cases, 10.0%), neurological events (9 cases, 6.0%), and others (2 cases, 1.3%).

Overall diagnostic performance of different information sources

Among the 150 autopsy-confirmed cases, the integration of clinical history and postmortem CT demonstrated superior diagnostic performance compared with either information source alone. For underlying causes of death, the integrated approach achieved 78.0% accuracy for primary diagnoses, which was significantly higher than that for clinical history alone (69.3%) or CT alone (42.0%) (p<0.001; Table 1). This hierarchical pattern was consistent across both primary diagnoses and when including the top three differential diagnoses.

For immediate causes of death, a similar but less pronounced pattern was observed. The integrated approach achieved significantly higher accuracy for primary diagnoses compared with clinical history alone and CT alone (p<0.001). When considering the top three differential diagnoses, the accuracy improved across all modalities (p=0.002) (Table 1).

**Table 1:** Diagnostic accuracy of different information sources in determining the causes of death Values are presented as % (n/N) †Cochran’s Q test across the three information sources ‡Post-hoc analysis using McNemar’s test with the Bonferroni correction (p<0.0167) a, clinical history vs. CT findings significant; b, CT findings vs. integrated information significant; c, clinical history vs. integrated information significant; CT, computed tomography

Analysis category	Clinical history only	CT findings only	Integrated information	Cochran's Q (df=2)	p-value†	Post-hoc analysis‡
Underlying causes of death						
Primary diagnosis	69.3 (104/150)	42.0 (63/150)	78.0 (117/150)	45.49	<0.001	a (χ²=19.32), b (χ²=33.52), c (χ²=3.67)
Top three diagnoses	78.0 (117/150)	58.7 (88/150)	84.7 (127/150)	28.28	<0.001	a (χ²=13.56), b (χ²=24.53), c (χ²=3.03)
Immediate causes of death						
Primary diagnosis	52.0 (78/150)	46.7 (70/150)	61.3 (92/150)	6.64	<0.001	b (χ²=6.05), c (χ²=2.72)
Top three diagnoses	74.0 (111/150)	72.0 (108/150)	82.0 (123/150)	4.61	0.002	b (χ²=5.36), c (χ²=3.69)

Disease-specific diagnostic accuracy analysis of the underlying causes of death

The disease-specific diagnostic accuracy analysis revealed marked variations in the diagnostic accuracy across different categories (Table 2). Among the malignant neoplasms, which comprised the largest group (79 cases, 52.7%), hematologic malignancies showed the most striking differences among the information sources, with the integrated approach achieving significantly higher accuracy (p=0.003). GI tract and hepatobiliary malignancies demonstrated similar patterns of improvement with the integrated approach (p<0.05).

**Table 2:** Disease category-specific analysis of diagnostic accuracy for the underlying causes of death Values are presented as % (n/N) or n (%) †Cochran’s Q test across the three information sources ‡Statistically significant after the Bonferroni correction (p<0.0167) ∫Including breast cancer (n=2) *Including leukemia, lymphoma, and multiple myeloma **Including esophageal, gastric, and colorectal cancers §Including autoimmune/inflammatory diseases, metabolic/systemic diseases, and other miscellaneous conditions CT, computed tomography

Cause-of-death category	N (%)	Clinical history only	CT findings only	Integrated information	Cochran's Q (df=2)	p-value†
Malignant neoplasms∫						
Hematologic malignancy*	28 (18.7)	78.9 (22/28)	36.8 (10/28)	85.7 (24/28)	12.29	0.003‡
Gastrointestinal tract cancer**	16 (10.7)	70.8 (11/16)	45.8 (7/16)	81.3 (13/16)	3.4	0.042
Hepatobiliary cancer	13 (8.7)	70.0 (9/13)	40.0 (5/13)	80.0 (10/13)	3.03	0.045
Lung cancer	11 (7.3)	73.3 (8/11)	53.3 (6/11)	86.7 (9/11)	1.34	0.137
Urologic/gynecologic cancer	9 (6.0)	71.4 (6/9)	42.9 (4/9)	85.7 (8/9)	2.67	0.135
Cardiovascular disease						
Cardiomyopathy/chronic heart failure	17 (11.3)	76.2 (13/17)	47.6 (8/17)	85.7 (15/17)	4.91	0.021
Aortic disease	7 (4.7)	77.8 (5/7)	66.7 (4/7)	88.9 (6/7)	0.67	0.549
Other major categories						
Liver disease	16 (10.7)	75.0 (12/16)	56.3 (9/16)	87.5 (14/16)	3.71	0.028
Respiratory disease	7 (4.7)	71.4 (5/7)	57.1 (4/7)	71.4 (5/7)	0.67	0.717
Neurologic disease	11 (7.3)	72.7 (8/11)	72.7 (8/11)	72.7 (8/11)	0	1
Others§	13 (8.7)	53.8 (7/13)	30.8 (4/13)	61.5 (8/13)	3.71	0.028
Total	150 (100)	69.3 (104/150)	42.0 (63/150)	78.0 (117/150)	45.49	<0.001

In cardiovascular diseases, cardiomyopathy/chronic heart failure cases demonstrated significant improvement with the integrated approach (p=0.021), while aortic diseases showed consistently high accuracy across information sources without reaching statistical significance. Analyses of liver diseases revealed that the integrated approach achieved significantly higher accuracy (p=0.028), while other categories showed improvements that did not attain statistical significance.

Disease-specific diagnostic accuracy analysis of immediate causes of death

Disease-specific diagnostic accuracy analyses of immediate causes revealed distinct patterns of diagnostic accuracy across different categories (Table 3). Respiratory failure showed varying accuracies across its subcategories, with pneumonia/infection and tumor-related respiratory failure demonstrating the most significant improvement with the integrated approach (p<0.05). The other respiratory subcategories showed modest improvements that did not reach statistical significance.

**Table 3:** Disease category-specific analysis of diagnostic accuracy for the immediate causes of death Values are presented as % (n/N) or n (%) †Cochran’s Q test across the three information sources

Cause-of-death category	N (%)	Clinical history only	CT findings only	Integrated information	Cochran's Q (df=2)	p-value†
Respiratory failure						
Pneumonia/infection	38 (25.3)	55.3 (21/38)	52.6 (20/38)	68.4 (26/38)	1.5	0.011
Tumor-related	14 (9.3)	64.3 (9/14)	57.1 (8/14)	71.4 (10/14)	0.41	0.042
Interstitial pneumonia/diffuse alveolar damage	11 (7.3)	54.5 (6/11)	45.5 (5/11)	63.6 (7/11)	0.49	0.165
Neuromuscular/airway	5 (3.3)	40.0 (2/5)	40.0 (2/5)	60.0 (3/5)	1	0.368
Others	5 (3.3)	40.0 (2/5)	40.0 (2/5)	60.0 (3/5)	1	0.368
Circulatory failure						
Heart failure/cardiogenic shock	18 (12.0)	55.6 (10/18)	44.4 (8/18)	66.7 (12/18)	1.2	0.097
Hemorrhagic shock	7 (4.7)	42.9 (3/7)	42.9 (3/7)	57.1 (4/7)	1	0.368
Multiple organ failure	22 (14.7)	45.5 (10/22)	40.9 (9/22)	54.5 (12/22)	0.57	0.264
Hepatic failure/gastrointestinal complications	15 (10.0)	46.7 (7/15)	40.0 (6/15)	53.3 (8/15)	1	0.368
Neurologic events	9 (6.0)	44.4 (4/9)	33.3 (3/9)	55.6 (5/9)	0.67	0.717
Total	150 (100)	52.0 (78/150)	46.7 (70/150)	61.3 (92/150)	6.64	<0.001

Circulatory failure, multiple organ failure, and hepatic failure/gastrointestinal complications showed relatively lower accuracies across all information sources, with modest improvements using the integrated approach that did not reach statistical significance.

Analysis of the diagnostic agreement between clinical history and CT findings

Analyses of the diagnostic agreement between the clinical history and CT findings revealed significant differences between the underlying and immediate causes of death (Table 4). The agreement between the clinical history and CT findings was notably higher for underlying causes (72.0%) than for immediate causes (47.3%; p<0.001). The proportion of cases in which neither source was correct was significantly higher for immediate causes (27.3%) than for underlying causes (9.3%; p<0.001), while the proportion of cases in which only one source was incorrect did not differ significantly between underlying and immediate causes. Disease-specific analyses revealed distinct patterns across diagnostic categories, with the highest rates of complete agreement observed in hematologic malignancy and heart disease for underlying causes and in pneumonia/infection and tumor-related multiple organ failure for immediate causes. Diagnostic failures were more frequently observed in multiple organ failure and neurological events, reflecting the inherent complexity of determining terminal events in these conditions.

**Table 4:** Analysis of diagnostic agreement between clinical history and CT findings in determining the causes of death (N=150) Values are presented as n (%) All analyses considered diagnoses among the three top differential diagnoses †The chi-square test or Fisher’s exact test comparing proportions between underlying and immediate causes The overall chi-square value for comparison across all patterns is 161.33 (df=3), p<0.001 CT, computed tomography

Performance pattern	Underlying cause	Immediate cause	Chi-square (df=1)	p-value†
Agreement pattern				
Clinical history and CT findings both correct	108 (72.0%)	71 (47.3%)	18.96	<0.001
Discordant pattern				
Clinical history correct, CT findings incorrect	16 (10.7%)	24 (16.0%)	1.85	0.215
CT findings correct, clinical history incorrect	12 (8.0%)	14 (9.3%)	0.17	0.837
Diagnostic failure				
Both sources incorrect	14 (9.3%)	41 (27.3%)	16.23	<0.001

## Discussion

This study provides a comprehensive evaluation of LLM performance in determining causes of death by comparing three different information sources (clinical history alone, postmortem CT findings alone, and their integration). Our analysis builds upon our previous work in this area [14] by examining how the integration of multiple information sources affects diagnostic accuracy across different disease categories. Our analysis revealed several key findings with important implications for clinical practice.

First, integrating the clinical history and postmortem CT findings demonstrated superior diagnostic performance over either source alone. This improvement was particularly evident for the underlying causes, with a similar but less pronounced pattern for the immediate causes. The value of considering multiple differential diagnoses highlighted the importance of maintaining broader diagnostic perspectives in death investigations.

Second, disease-specific analyses revealed striking variations in the utility of different information sources. For the underlying causes, hematologic malignancies showed the most pronounced differences among the information sources, reflecting the challenge of identifying these conditions through imaging alone. Similar patterns in the GI tract and hepatobiliary malignancies suggest that the clinical history is particularly crucial for the cancer diagnosis. Within cardiovascular diseases, aortic disorders showed a balanced utility of information sources, while cardiomyopathy/chronic heart failure demonstrated the greater utility of the patient’s clinical history.

Third, the marked disparity in the diagnostic agreement between the underlying and immediate causes warrants attention [16]. The higher rate of agreement between information sources for underlying causes suggests fundamental differences in the diagnostic challenges these determinations present. This disparity was particularly evident in complex terminal events, with multiple organ failure and neurological events showing consistently lower accuracy across all information sources. The substantial gains seen when considering multiple differential diagnoses for immediate causes suggest value in maintaining broader diagnostic considerations for terminal events.

Fourth, our analyses of disease-specific performance patterns provide insights for optimizing diagnostic strategies. The success of the integrated information was most evident in hematologic malignancies and cardiovascular diseases. Certain categories showed high accuracy with specific information sources, indicating the potential for streamlined assessment protocols. Conversely, the consistently lower accuracy in neurologic events and multiple organ failure highlights areas requiring enhanced diagnostic approaches. Building upon our previous work with 100 cases [14], this expanded study provides more comprehensive insights into these disease-specific variations.

Fifth, the LLM demonstrated distinct performance features that merit consideration. Its superior performance in underlying cause determination aligns with the more definitive nature of these conditions and their clearer documentation in clinical histories. The challenges in immediate cause determination reflect the difficulty in interpreting terminal events from fragmented information sources. This suggests that LLM performance might be enhanced through specifically tailored prompting strategies for different diagnostic categories.

Our findings have direct implications for clinical practice. The superior performance of the integrated approach supports its adoption as a standard practice in death investigation. However, the varying benefits across disease categories indicate the need for tailored diagnostic strategies. The optimization of resource allocation in death investigation might be guided by suspected disease categories, with some conditions requiring comprehensive assessment while others might be adequately evaluated with focused approaches.

Nevertheless, this study had several limitations that warrant consideration. As a single-center study using only one LLM, our findings may not fully generalize to other settings [12]. Our study population consisted exclusively of in-hospital deaths at a tertiary care center that underwent pathological autopsy, potentially introducing selection bias. Such cases often involve complex medical conditions with multiple comorbidities and complicated terminal courses. Sample sizes for some disease categories were limited. Furthermore, although we maintained the original Japanese format of medical histories and CT reports, language-specific variations in LLM performance cannot be excluded.

Additionally, our study does not address the medico-legal validity of LLMs in determining causes of death for forensic or legal purposes. Despite the promising performance shown in our research, significant challenges remain before such technology could be considered in legal contexts. These include the lack of explainability in AI decisions, current legal requirements for qualified medical professionals to certify deaths, and the complex nature of forensic determinations that often require professional judgment beyond pattern recognition. Future research should explore potential auxiliary roles for these technologies within established legal frameworks for death investigation while recognizing that LLMs should supplement rather than replace forensic pathologists' expertise.

Future research should address these limitations through multicenter studies with larger sample sizes. Investigations of additional information sources, such as laboratory data or specialized imaging techniques, could further enhance the diagnostic accuracy. The optimization of LLM prompting strategies, particularly for challenging categories such as immediate causes of death and complex terminal events, merits further exploration.

The potential role of LLMs in supporting death investigations deserves particular attention given the growing shortage of specialists in this field [17,19]. Our findings suggest that LLMs, when properly integrating multiple information sources, could serve as valuable diagnostic support tools. However, the varying accuracy across different disease categories and the particular challenges in determining immediate causes emphasize that these tools should complement expert judgment and not replace it.

## Conclusions

This study demonstrates that integrating clinical history with postmortem CT significantly improves LLM-based cause-of-death determination, although the benefit varies substantially by disease category. The marked differences in diagnostic accuracy between underlying and immediate causes, along with the disease-specific variations in information source utility, highlight the need for tailored diagnostic approaches. These findings support an integrated approach to investigating death while underscoring the importance of maintaining broad differential diagnoses, particularly for immediate causes of death.

## References

[1] Sonoda Y, Kurokawa R, Hagiwara A (2024). Structured clinical reasoning prompt enhances LLM’s diagnostic capabilities in diagnosis please quiz cases. Jpn J Radiol.

[2] Harigai A, Toyama Y, Nagano M (2025). Response accuracy of GPT-4 across languages: insights from an expert-level diagnostic radiology examination in Japan. Jpn J Radiol.

[3] Suzuki K, Yamada H, Yamazaki H, Honda G, Sakai S (2025). Preliminary assessment of TNM classification performance for pancreatic cancer in Japanese radiology reports using GPT-4. Jpn J Radiol.

[4] Sonoda Y, Kurokawa R, Nakamura Y (2024). Diagnostic performances of GPT-4o, Claude 3 Opus, and Gemini 1.5 Pro in "Diagnosis Please" cases. Jpn J Radiol.

[5] Kurokawa R, Ohizumi Y, Kanzawa J (2024). Diagnostic performances of Claude 3 Opus and Claude 3.5 Sonnet from patient history and key images in radiology's "Diagnosis Please" cases. Jpn J Radiol.

[6] Oura T, Tatekawa H, Horiuchi D (2024). Diagnostic accuracy of vision-language models on Japanese diagnostic radiology, nuclear medicine, and interventional radiology specialty board examinations. Jpn J Radiol.

[7] Hirano Y, Hanaoka S, Nakao T (2024). GPT-4 Turbo with vision fails to outperform text-only GPT-4 Turbo in the Japan Diagnostic Radiology Board Examination. Jpn J Radiol.

[8] Toyama Y, Harigai A, Abe M, Nagano M, Kawabata M, Seki Y, Takase K (2024). Performance evaluation of ChatGPT, GPT-4, and Bard on the official board examination of the Japan Radiology Society. Jpn J Radiol.

[9] Nakaura T, Yoshida N, Kobayashi N (2024). Preliminary assessment of automated radiology report generation with generative pre-trained transformers: comparing results to radiologist-generated reports. Jpn J Radiol.

[10] Asari Y, Kurokawa R, Sonoda Y (2024). "This is a quiz" premise input: a key to unlocking higher diagnostic accuracy in large language models. Cureus.

[11] Ueda D, Mitsuyama Y, Takita H, Horiuchi D, Walston SL, Tatekawa H, Miki Y (2023). ChatGPT's diagnostic performance from patient history and imaging findings on the diagnosis please quizzes. Radiology.

[12] Busch F, Hoffmann L, Dos Santos DP (2025). Large language models for structured reporting in radiology: past, present, and future. Eur Radiol.

[13] Akinci D'Antonoli T, Stanzione A, Bluethgen C (2024). Large language models in radiology: fundamentals, applications, ethical considerations, risks, and future directions. Diagn Interv Radiol.

[14] Ishida M, Gonoi W, Nyunoya K (2024). Diagnostic performance of GPT-4o and Claude 3 Opus in determining causes of death from medical histories and postmortem CT findings. Cureus.

[15] Inai K, Noriki S, Kinoshita K (2016). Postmortem CT is more accurate than clinical diagnosis for identifying the immediate cause of death in hospitalized patients: a prospective autopsy-based study. Virchows Arch.

[16] Roberts IS, Benamore RE, Benbow EW (2012). Post-mortem imaging as an alternative to autopsy in the diagnosis of adult deaths: a validation study. Lancet.

[17] Rutty GN, Morgan B, Robinson C (2017). Diagnostic accuracy of post-mortem CT with targeted coronary angiography versus autopsy for coroner-requested post-mortem investigations: a prospective, masked, comparison study. Lancet.

[18] Shelmerdine SC, Davendralingam N, Palm L, Minden T, Cary N, Sebire NJ, Arthurs OJ (2019). Diagnostic accuracy of postmortem CT of children: a retrospective single-center study. AJR Am J Roentgenol.

[19] Lefèvre T, Tournois L (2023). Artificial intelligence and diagnostics in medicine and forensic science. Diagnostics (Basel).

